# Can Plants Grow on Mars and the Moon: A Growth Experiment on Mars and Moon Soil Simulants

**DOI:** 10.1371/journal.pone.0103138

**Published:** 2014-08-27

**Authors:** G. W. Wieger Wamelink, Joep Y. Frissel, Wilfred H. J. Krijnen, M. Rinie Verwoert, Paul W. Goedhart

**Affiliations:** 1 Alterra, Wageningen UR, Wageningen, the Netherlands; 2 Unifarm, Wageningen UR, Wageningen, the Netherlands; 3 Biometris, Wageningen UR, Wageningen, the Netherlands; Leibniz-Institute for Farm Animal Biology (FBN), Germany

## Abstract

When humans will settle on the moon or Mars they will have to eat there. Food may be flown in. An alternative could be to cultivate plants at the site itself, preferably in native soils. We report on the first large-scale controlled experiment to investigate the possibility of growing plants in Mars and moon soil simulants. The results show that plants are able to germinate and grow on both Martian and moon soil simulant for a period of 50 days without any addition of nutrients. Growth and flowering on Mars regolith simulant was much better than on moon regolith simulant and even slightly better than on our control nutrient poor river soil. Reflexed stonecrop (a wild plant); the crops tomato, wheat, and cress; and the green manure species field mustard performed particularly well. The latter three flowered, and cress and field mustard also produced seeds. Our results show that in principle it is possible to grow crops and other plant species in Martian and Lunar soil simulants. However, many questions remain about the simulants' water carrying capacity and other physical characteristics and also whether the simulants are representative of the real soils.

## Introduction

Lunar and Mars explorations have provided information about the mineral composition of the soils of these solar objects. In addition to rocks they contain large amounts of sand-like soils or regoliths. All essential minerals for the growth of plants appear to be present in sufficient quantities in both soils probably with the exception of reactive nitrogen. Nitrogen in reactive form (NO_3_, NH_4_) is one of the essential minerals necessary for almost all plant growth [1]. The major source of reactive nitrogen on Earth is the mineralisation of organic matter [1]. However organic matter is absent on both Mars and moon although they do contain carbon [2]–[6]. Nitrogen in reactive form (NO_3_, NH_4_) is one of the essential minerals necessary for almost all plant growth [1]. Reactive nitrogen is part of the material in our solar system and is part of solar wind, a source of reactive nitrogen on the moon and Mars [3], [7]. Reactive nitrogen may also arise as an effect of lightning or volcanic activity [8], [9] and both processes may occur on Mars. This indicates that in principle reactive nitrogen could be present [7], [10]. However, the Mars Pathfinder was not able to detect reactive nitrogen [11]. Thus the actual presence of major quantities of reactive nitrogen remains uncertain. The major source of reactive nitrogen on Earth is the mineralisation of organic matter [1], which is absent on both Mars and moon. The absence of sufficient reactive nitrogen may be solved by using nitrogen fixing species. In symbioses with bacteria [12], [13] these nitrogen fixers are able to bind nitrogen from the air and transform it into nitrates, a process which requires nitrogen in the atmosphere. However, there is no atmosphere on the moon, and on Mars it is only minimally present and contains traces of nitrogen. Metals like aluminium and chromium are also present in the extra-terrestrial soils. Aluminium is known to disturb plant growth and even lead to plant death [14]. Another essential for plant growth is liquid water. Liquid water is not (moon) or possibly very limited present (Mars). Ice is present on both Mars and moon, and could be used after harvest [15]–[17]. Many plant species may be grown on water cultures, e.g. tomatoes or paprika, but not all. Therefore, local soils could be used to grow crops, at least partly.

During the Apollo project there has been no experiment with plant growth on the moon. However experiments on earth have been carried out with the brought back moon material. These experiments did not include growth of plants on moon soil. Instead plants were exposed to moon stones by rubbing them and even small amounts were added to growth medium. These experiments indicated that there were no toxic effects of moon soil on short term plant growth [18], for an overview see Ferl and Paul [19]. Ferl and Paul [19] also provide pictures of the model plant *Arabidopsis thaliana* grown on a moon regolith simulant (JSC1a). Studies with moon rock simulant (anorthosite) were carried out with the model plant *Tagetes patula* [20], [21]. These studies revealed that these plants were able to grow with and without the addition of bacteria [20], [21], and that plants were able to blossom [20]. There have been plant growth experiments with Mars regolith simulant as well. Experiments with bacteria on Mars soil simulant revealed that growth is possible, including nitrogen fixing bacteria [22].

Our goal was to investigate whether or not species of the three groups wild plants, crops and nitrogen fixers (Table 1), would germinate and live long enough to go through the first stages of plant development on artificial Mars and moon regoliths. If this would be the case it is conceivable that plant growth is possible within an artificial surrounding on Mars and moon surface, although our experiment was conducted on Earth with its deviating gravity. Moreover, we assumed that plant cultivation will be carried out in closed surroundings with Earth like light and atmospheric conditions.

**Table 1:** Species used in the experiment, the species group it belongs to and information about the species trait partly based on Wamelink et al. [29], [30], [36].

Latin	English	Group	Abbreviation	description
*Arnica montana*	Leopards bane	Occurring naturally	ARM	Species of nutrient poor dry soil conditions with a light acidic pH.
*Sinapsis arvensis*	Field mustard	Occurring naturally	SIA	Species of nutrient rich soil conditions. Often used as green manure in winter.
*Urtica dioica*	Stinging nettle	Occurring naturally	URD	Ruderal species, can become dominant under nutrient rich soil conditions, mostly on soils with a light acidic till basic pH.
*Cirsium palustre*	Marsh thistle	Occurring naturally	CIP	Ruderal species, can become dominant under nutrient rich soil conditions, mostly on soils with a light acidic till basic pH.
*Sedum reflexum*	Reflexed stonecrop	Occurring naturally	SER	Species of (extreme) nutrient poor (extreme) dry soil conditions, mostly on soils with a light acidic till basic pH.
*Festuca rubra*	Red fescue	Occurring naturally	FER	Gras species that can withstand many circumstances from nutrient poor acidic dry till nutrient rich basic moist conditions.
*Vicia sativa sativa*	Common vetch	Nitrogen fixer	VIS	Species used as green manure or livestock fodder and eatable for humans. Cattle feed faster on vetch than on most grasses.
*Lupinus angustifolius*	Lupin	Nitrogen fixer	LUA	Known of soil improvement and is used as green manure or as a grain legume for human consumption or animal feed.
*Melilotus officinalis*	Yellow sweet clover	Nitrogen fixer	MEO	Biannual species that likes basic soils and is drought resistant. It does not like shaded places.
*Lotus pedunculatus*	Greater birds'-foot trefoil	Nitrogen fixer	LOP	Moist loving species of light acidic till neutral modest nutrient rich soils
*Solanum lycopersicum*	Tomato	Crop	SOL	The Tomato can be grown as an annual or perennial. It likes light acidic till basic soils that can be dry till wet.
*Secale cereale*	Rye	Crop	SEC	The seeds of Rye can be used for many eatable products. It is able to grow at relative low temperatures (winter hardy) and can grow in nutrient poor light acidic till basic dry soils.
*Daucus carota s. sativus*	Carrot	Crop	DAC	Biannual species, that likes sunny places and moist light acidic till basic not to nutrient rich soils.
*Lepidium sativum*	Garden cress	Crop	LES	Fast growing species that likes moist circumstances, but is known to grow almost anywhere.

## Materials and Methods

### Regoliths

Mars and moon regolith simulant were purchased from Orbitec (http://www.orbitec.com). Both regoliths were manufactured by NASA (for Mars we used JSC-1A Mars regolith simulant, for Moon we used the JSC1-1A lunar regolith simulant) [23], [24]. Since the Mars and moon regolith simulants are comparable to Earth soils, at least in mineral composition [23]–[28], they can be mimicked by using volcanic Earth soils, as has been done by NASA [23], [24].

As a control we used coarse river Rhine soil from 10 m deep layers which is nutrient poor, and free from organic matter and seeds. Since the moon and Mars simulants had only been analysed for mineral content and particle size, we also analysed them for nutrients that are available for plant species. All three soil types were analysed for soil pH water, Organic matter content, Total N and P content (both destructive), NH_4_, NO_2_+, NO_3_, PO_4_, Al, Fe, K and Cr (all seven in CaCl_2_ extract). All analyses were repeated two times according to standard protocol (RvA-accreditation for test laboratories; registration number scope: 342). These soil parameters are typically used to explain species occurrence on Earth [29].

The analysis revealed that the moon regolith simulant is truly nutrient poor, though it contains a small amount of nitrates and ammonium. The Mars regolith simulant also contains traces of nitrates of ammonium, and also a significant amount of carbon (Table 2). The pH of all three soils is high. The pH of the moon regolith is that high that it may be problematic for many plant species, especially for crops [30]. We applied the regoliths and the control earth sand as supplied, the sands were not sterilised, since sterilisation may alter its properties.

**Table 2:** Analyses of the soil samples, in red the detection limits of the analysis.

Method		SFA-Nt/Pt destruction with H_2_SO_4_-H_2_O_2_-Se		ICP-AES extraction in 0.01 M CaCl_2_			ICP-MS	SFA extraction with 0,01M CaCl_2_			pH-H_2_O	LECO-CHN		CaCO_3_
Element		Nt	Pt	Al	Fe	K	Cr	N-NH4	N-(NO_3_+NO_2_)	P-PO4	at 20±1°C	C-elementary	N-elementary	Scheibler
Unit		[g/kg]	[mg/kg]	[mg/kg]	[mg/kg]	[mg/kg]	[µg/kg]	[mg/kg]	[mg/kg]	[mg/kg]	-	[g/kg]	[g/kg]	[%]
detection limit		0.3	100.0	0.5	3.0	3.0	5.0	1.0	0.5	0.4	-	3.0	0.3	
average	Earth	0.0	57.3	0.0	0.0	4.7	2.0	0.5	4.2	0.0	8.3	3.2	0.0	
	moon	0.0	1003.0	0.5	0.0	27.0	0.0	0.3	4.2	0.2	9.6	3.0	0.0	
	Mars	2.6	2487.7	0.0	0.0	138.0	0.0	3.9	2.1	0.0	7.3	30.1	2.5	0.2
s.e	Earth	0.2	1.5	0.2	0.0	0.6	3.5	0.1	0.2	0.0	0.0	0.1	0.1	
	moon	0.2	11.0	0.1	0.0	0.0	0.0	0.1	0.1	0.0	0.0	0.1	0.1	
	Mars	0.1	28.4	0.2	0.0	1.0	0.6	0.1	0.0	0.0	0.0	0.5	0.1	0.0

### Species selection

Species were selected from three groups: four different crops, four nitrogen fixers and six wild plants which occur naturally in the Netherlands (Table 1). Only species with relatively small seeds were chosen so that the nutrient stock in the seeds would be quickly depleted and the plant becomes totally dependent on what is available in the soils for its growth. For the wild plants we chose species that are able to grow either under nutrient poor circumstances or under a wide range of circumstances (see Table 1) based on the responses of the species to abiotic conditions [29], [30]. Note that although species may have limits for growth conditions in the field they are often able to grow in monocultures under different circumstances, e.g. more nutrient rich or nutrient poor conditions, because of lack of more competitive species. To be able to monitor the first growth stages we used seeds of the species. The crop and nitrogen fixer seed were bought at the local shop (Welkoop, Wageningen), and the wild plant seeds at Cruydt Hoeck (Nijeberkoop). The latter seeds were collected in the field. Externally present bacteria on the seeds, if any, were not killed.

### Experimental design and observations

**Figure 1:**
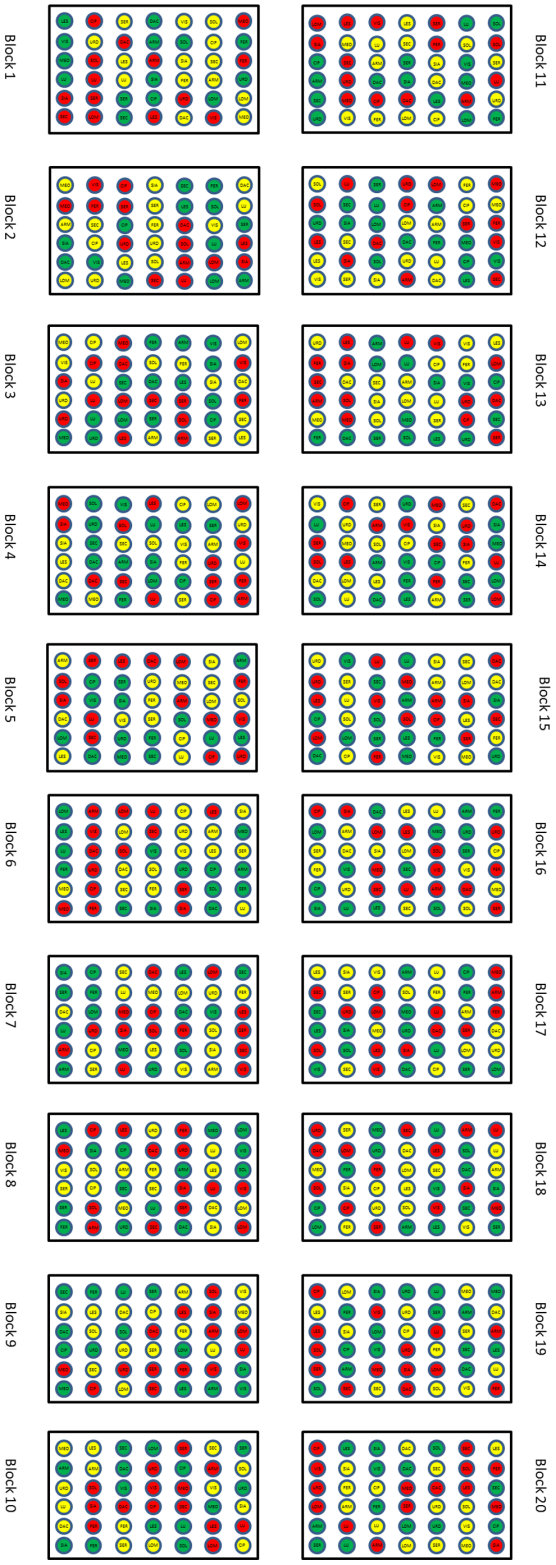
Design of the experiment with the first ten blocks the west oriented part of the experiment and the second ten blocks the east oriented part of the experiment. For abbreviations of the species see Table 1.

**Figure 2:**
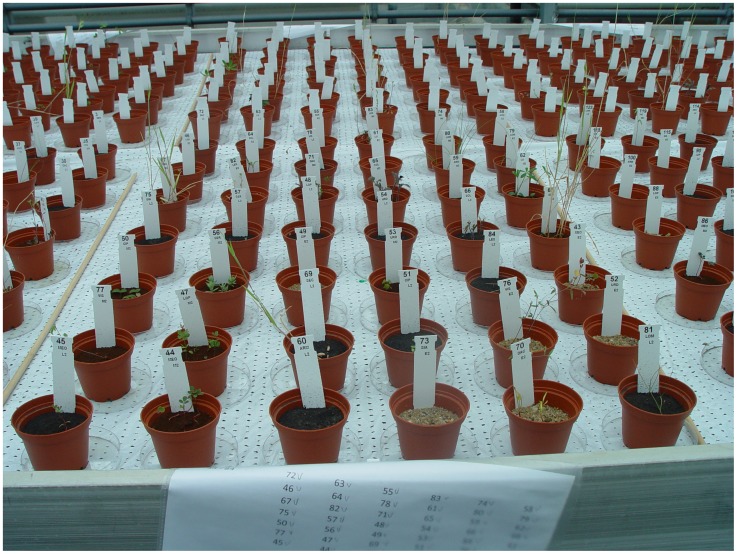
Block 2 of the experiment, with randomly placed pots, 14 days after the start of the experiment. Each block contains 42 pots. Block 12 is visible in the background. The labels in the pots show the pot number, the species (from left to right on the first row Yellow sweet clover (twice), Leopards bane, Field Mustard, Carrot and Red fescue) and the soil type (L for moon or Lunar, M for Mars and E for Earth) combined with the block number (2).

Small pots were filled with 100 g moon soil simulant, 100 g Earth soil or 50 g Mars soil simulant and 25 g demineralized water was added to each pot. The mass of the simulants added was different since we wanted to fill the pots with approximately the same volume to have the same column height. A filter was placed on the bottom of each pot to prevent soil from leaking. For each soil type and plant species twenty replica pots were used. This resulted in 840 pots (3 soils×14 species×20 replicas). In each pot we positioned five seeds, giving 100 seeds per species - soil combination. The pots were placed in a glasshouse in a completely randomized block design where each block constitutes a replicate (Fig. 1). Each pot was placed in a petri dish (without cap) to hold excessive water and to prevent roots growing into other pots. The pots were placed on a large table in the glasshouse (Fig. 2).

The experiment started of April 8^th^ 2013. Temperature in the glasshouse was maintained at around 20°C. During the experimental period average temperature was 21.1±3.02°C and air humidity was 65.0±15.5% both based on 24 hour recording with a 5 minutes interval. Mean day time lasted for 16 hours. If the sunlight intensity was below 150 watt/m^2^ lamps yielding 80 µmol (HS2000 from Hortilux Schréder) were switched on. The pots were watered once or twice a day depending on the evaporation rate by spraying with demineralised water (about 10 litres for the whole experiment for each occasion). We used demineralized water to mimic water from Mars and moon and to prevent pollution with (for example) nutrients that are present in tap water. Ambient air was used.

Seeds were scored on germination, first leaf production, bud forming, flowering and seed setting. At the end of the experiment, 50 days after April 8^th^, total biomass was harvested and, after cleaning, dried in a stove for 24 hours at 70°C; After cooling down above and below ground biomass were weighed separately. For 25 experimental units the total biomass was smaller than the weighting limit. For those units a value of 0.5 mg (for plants that germinated, but could not be recovered at the end of the experiment) or 0.1 mg (for plants that died before the end of the experiment directly after germination) was assigned to the total biomass. Above and below ground biomass was set to half this value. For 21 units the above ground biomass was smaller than the weighting limit and this was also true for the below ground biomass of 25 units. In these cases the corresponding biomass was set to 0.1 mg.

### Statistical Analysis

Logistic regression was used to statistically analyse the number of germinated seeds in each pot, as well as the number of seeds which developed leaves, which developed flowers (including buds), and the numbers of plants which were still alive after 50 days. A pairwise likelihood ratio test, separately for each species and accounting for differences between blocks, was employed to test whether Earth, moon and Mars soil simulants give different results. When necessary, overdispersion was accounted for by inflating the binomial variance by an unknown factor and then using quasi likelihood rather than maximum likelihood [31].

An analysis of variance, again separately for each species and accounting for block effects, was performed on the logarithm of the total, above and below ground biomass, as well as on the ratio of the above and below ground biomass. The log transform was employed because this stabilizes the variance. Pairwise difference t-test between the soil types were carried out. Note that this is a conditional analysis since units with no biomass are excluded. This implies that no biomass is given for *V. sativa sativa* on the moon because none of these seeds germinated.

## Results

**Figure 3:**
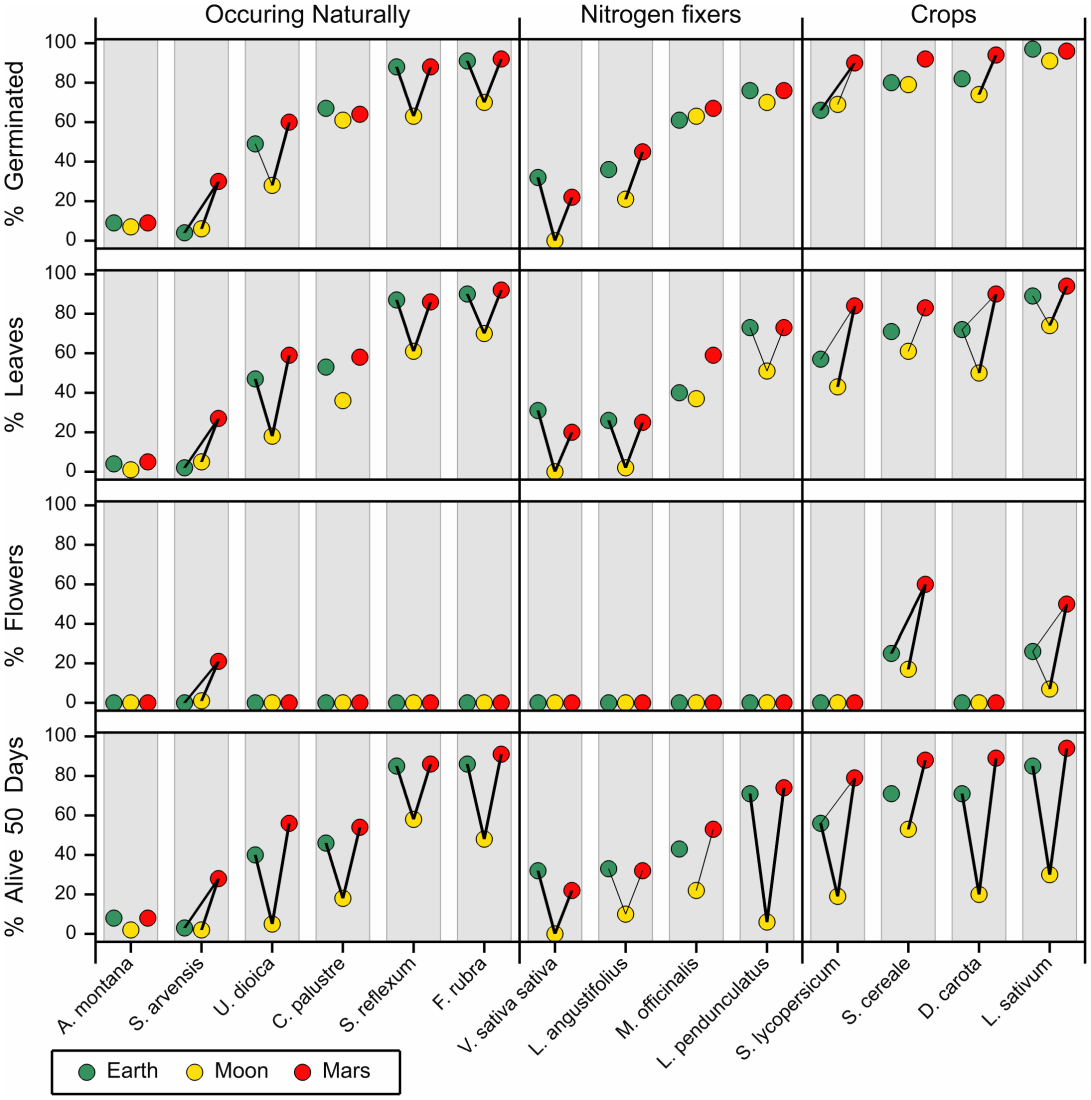
Percentage germination, leave formers, plants forming flowers and plants still alive after 50 days per species. All results are after 50 days and percentages are based on all 100 seeds per plant species-soil type combination Pairwise differences are displayed by a line which joins soil types which are significantly different at the 1% (thin line) and 0.1% (thick line) significance level. Background information can be found in Table S1 and S2.

Common vetch, a nitrogen fixer, did not germinate on moon soil. All other plant species did germinate with different proportions on all soils (Fig. 3; background information can be found in Table S1 and S2). In general the germination percentage on Martian soil simulant is highest and lowest on the moon soil simulant (Fig. 3). On average the four crop species have the highest germination percentages, although some species (Reflexed stonecrop, Red fescue, Yellow sweet clover and Greater birds'-foot trefoil) from the other two groups have similar germination percentages. Differences in germination percentages are most likely due to seed quality. The seeds of the crops Carrot, Cress and Tomato are controlled and have a high quality. The seeds of the other species are harvested from the field and except Rye have not been improved by plant breeding. These seed lots may therefore contain less or non-viable seeds. The percentages of plants that form leaves are sometimes considerably lower than the percentages for germination, indicating that some plants stop developing or even die. Leaf forming occurred most on Martian soil simulant and least on moon soil simulant. This trend is also present for species that form flowers or seeds. Only three species reach these stages, Field mustard, Rye and Cress (the last two being crops). Field mustard (only on Mars) and Cress (on Mars and Earth) also formed seeds. For examples see photo 1–10 (File S1). Also for the percentage plants still alive after 50 days, Martian soil simulant performed best and moon soil simulant worst. Martian soil simulant also performed better than Earth soil for most species. Leopards bane, Field mustard and Common vetch had no living plants left after 50 days on moon soil.

**Figure 4:**
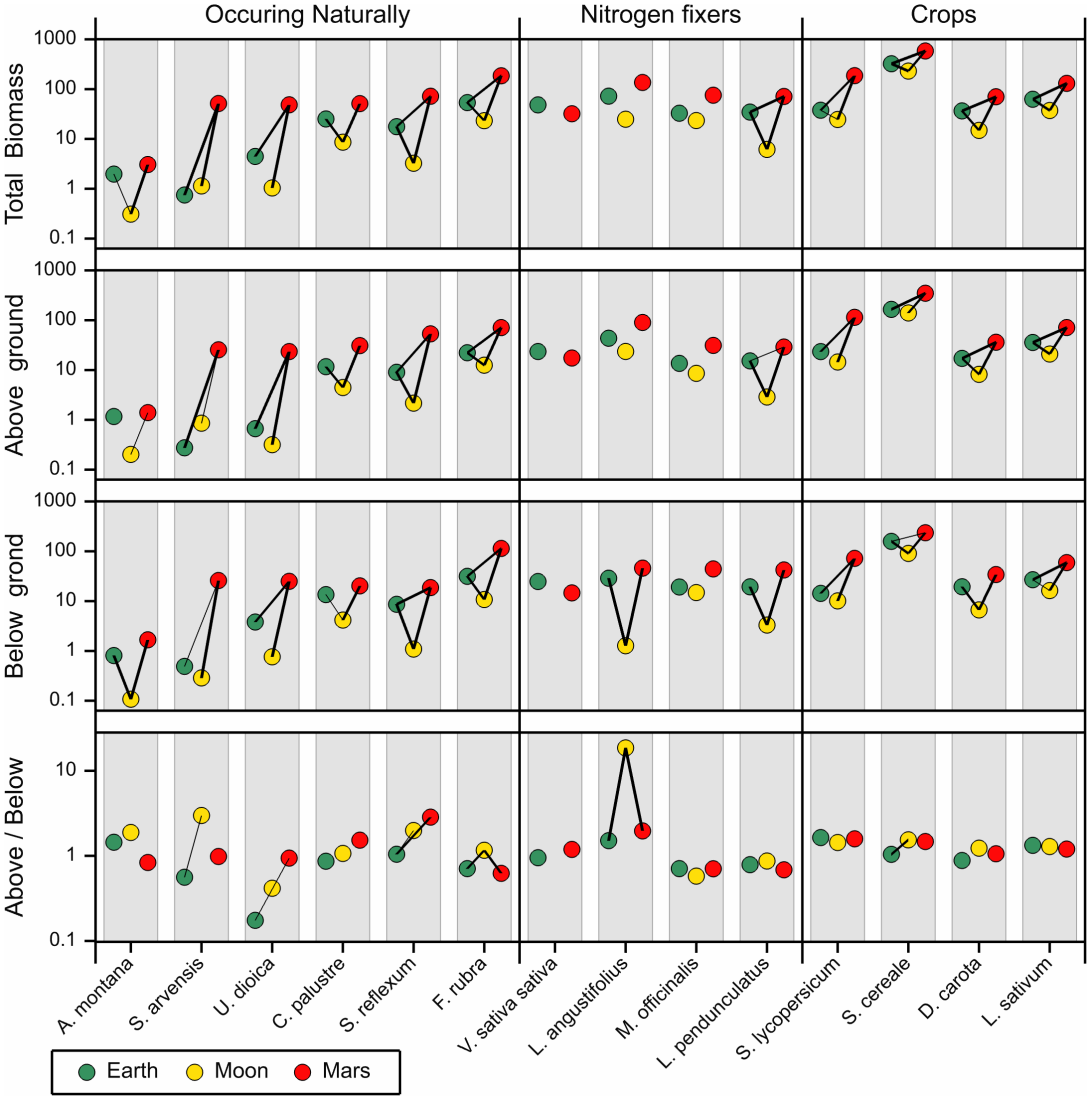
Average biomass results per species at the end of the 50 day experiment and the resulting aboveground belowground biomass ratio. Biomasses are given in mg dry weight on 10 log scale. The triangle indicates an outlier for Lupine (above/below 19.7). For Common vetch there is no ratio given because both above- and belowground biomass are zero. Pairwise differences are displayed by a line which joins soil types which are significantly different at the 1% (thin line) and 0.1% (thick line) significance level. Background information can be found in Table S1 and S2.

The biomass at the end of the experiment was significantly higher for eleven out of the fourteen species on Martian soil simulant as compared to both other soils. The biomass for earth and moon soil simulant is often quite similar (Fig. 4), although for nine species the biomass increment on Earth soil was significantly higher than on moon soil simulant. Apparently, in general, plants were able to develop at the same rate on Martian and Earth soil simulants, but biomass increment was much higher on Mars simulant. This is reflected in both below and aboveground biomass, although there are differences at the species level.

## Discussion

We found germination and plant growth for both moon and Mars soil simulants. Our results are in line with earlier research on *Arabidopsis thaliana* and *Tagetes patula* [19]–[21] on moon regolith simulant and moon rock simulant, though our results appear to be less promising. Kozyrovska et al. [20] had blossoming plants of *T. patula*, where we had only one plant of *Sinapsis arvensis* that formed a flower butt, but died before flowering.

On average species in Martian soil simulant performed significantly better than plants in Earth soil with respect to biomass increment. Although the Earth soil used, which was coarse and very nutrient poor, is not the best soil to grow crops on, we expected it to perform at least as well as the other two soils. However, in the warmer periods it was difficult to keep the water content in the pots high enough, despite spraying twice a day. The Mars soil simulant resembles loess-like soils from Europe and holds water better than the other two soils. Moon soil simulant dried out fastest. It therefore is essential that further research on the physical characteristics of the extra-terrestrial soils is conducted, as well as the way they could be irrigated. The larger water holding capacity of Martian soil simulant may explain its better performance and, partly, the underperformance of moon soil simulant. The high pH may also explain the lagging growth on the moon soil simulant and also on the Earth soil. Important for plant growth is not only the presence of nutrients, but also the balance between them. Both soils are rather imbalanced for nutrients; where the artificial moon soil lacks nitrates, the artificial soil lacks of phosphate. If nutrients are added in future experiments this imbalance has to be corrected as well, besides the addition of nutrients itself. The presence of a high C-elementary content in the Mars soil simulant is surprising. We also chemically analysed organic matter content in the simulant, but that resulted in obviously wrong results. The standard procedure includes backing the soil at 550°C. The problem is that part of the oxides, especially the iron oxides, evaporates as well, clearly yielding wrong results. Nevertheless a part of the origin of the Carbon content may be from organic matter. It may be a result of the way the soil is ‘harvested’ on Hawaii, leaving traces of organic carbon in the soil. Kral et al. [22] found traces of organic material in the JSC-1 simulant. It may also partly explain why the Mars soil was able to hold water best, as organic matter is more capable of holding water than bare sand. There is no organic matter on Mars [2]–[6], as far as we now, so this would make the Mars simulant we used less suitable for experiments to investigate the potential of Mars soil, unless the experiment has as goal to test the potential of the soil after adding organic matter. In our experiment this was the reason to test the legumes. They can be used as green fertilizer and after growth mixed with the soil. Visual inspection of the Mars soil simulant did not reveal large quantities of organic matter. However, further test on the simulant is advisable.

This experiment was carried out in pots. Some of the crops on Mars or moon may be cultivated in pots, but part of the crops may possibly be cultivated in full soil (in growth chambers or under domes). Moist conditions will then be different and may give rise to different results between pots and full soil. It is therefore of interest to conduct future experiments in full soil cultivation as well.

The reason for using nitrogen fixers in our experiment is that they may possibly compensate for the lack of sufficient reactive nitrogen in artificial Martian and moon soil. At the first stage of colonisation, these species can be used to enrich the soils with nitrogen, essential for all other plants, by mixing them with the soil after their growth as is commonly done in the Netherlands in winter [32]–[34]. This may be done in addition to manure brought from Earth or from human faeces. All chosen nitrogen fixers may perform this function; however Common vetch did not perform very well on Martian soil simulant, which may indicate that inoculation with nitrogen fixing bacteria may be necessary. We did not inoculate the soil simulants with nitrogen fixing bacteria in this experiment, although we did not sterilise the simulants nor the seeds. The bacteria could thus be present, but we did not test that in our experiment. In future experiments we will inoculate the soils with these bacteria. The nitrogen fixers may also play a role in detoxifying soils polluted with metals [35].

## Conclusions

Except for Common vetch all other plants germinated in some proportion on all three tested soils; the Mars soil simulant, the moon soil simulant and the River Rhine soil (control). Rye, cress and field mustard flowered, the latter two also formed seeds. Germination and biomass forming differed between species and soil types. The Mars soil simulant gave the highest biomass production, the moon soil simulant the lowest. On the moon soil simulant many germinated plants died or stayed very small. This may be due to the high soil pH, the moist holding capacity and or the free aluminium in the simulant. Our results show that it is in principle possible to grow plants in Martian and Lunar soil simulants although there was only one plant that formed a flower butt on moon soil simulant. Whether this extends to growing plants on Mars or the moon in full soils themselves remains an open question. More research is needed about the representativeness of the simulants, water holding capacity and other physical characteristics of the soils, whether our results extend to growing plants in full soil, the availability of reactive nitrogen on Mars and moon combined with the addition of nutrients and creating a balanced nutrient availability, and the influence of gravity, light and other conditions.

## Supporting Information

Table S1 Percentages seeds which germinated, produced leaves, were flowering and were alive after 50 days. P values of pairwise difference tests, separately for each species, are given in the last three columns. P-values smaller than 0.01 are given in bold. All species soil type combinations had 20 replicas and five seeds were positioned in every pot. Note that due to the many replicas small differences are statistically significant.
(DOCX)

Table S2 Number of seeds that Germinated, formed green leaves, flowered, set seeds, number of plants alive after 50 days, total biomass per pot, below ground biomass per pot and above ground biomass per pot. (see Excel file).
(XLSX)

File S1 **Photos of the experiment.**
(DOCX)
