# Supplementary material for: Can Plants Grow on Mars and the Moon: A Growth Experiment on Mars and Moon Soil Simulants
Source: PLoS One. 2014 Aug 27;9(8):e103138. doi: 10.1371/journal.pone.0103138 (PMC4146463; doi:10.1371/journal.pone.0103138)
Supplement: Table S1 — Percentages seeds which germinated, produced leaves, were flowering and were alive after 50 days. P values of pairwise difference tests, separately for each species, are given in the last three columns. P-values smaller than 0.01 are given in bold. All species soil type combinations had 20 replicas and five seeds were positioned in every pot. Note that due to the many replicas small differences are statistically significant. (DOCX) [file pone.0103138.s001.docx]

| **Germinated** | | | | | | |
| --- | --- | --- | --- | --- | --- | --- |
| Species | %Earth | %moon | %Mars | Earth-moon | Earth-Mars | moon-Mars |
| *A. montana* | 9 | 7 | 9 | 0.590 | 1.000 | 0.590 |
| *S. arvensis* | 4 | 6 | 30 | 0.511 | **0.000** | **0.000** |
| *U. dioica* | 49 | 28 | 60 | **0.005** | 0.138 | **0.000** |
| *C. palustre* | 67 | 61 | 64 | 0.426 | 0.687 | 0.692 |
| *S. reflexum* | 88 | 63 | 88 | **0.000** | 1.000 | **0.000** |
| *F. rubra* | 91 | 70 | 92 | **0.000** | 0.798 | **0.000** |
| *V. sativa sativa* | 32 | 0 | 22 | **0.000** | 0.096 | **0.000** |
| *L. angustifolius* | 36 | 21 | 45 | 0.020 | 0.187 | **0.001** |
| *M. officinalis* | 61 | 63 | 67 | 0.806 | 0.459 | 0.619 |
| *L. pendunculatus* | 76 | 70 | 76 | 0.311 | 1.000 | 0.311 |
| *S. lycopersicum* | 66 | 69 | 90 | 0.673 | **0.000** | **0.001** |
| *S. cereale* | 80 | 79 | 92 | 0.874 | 0.030 | 0.021 |
| *D. carota* | 82 | 74 | 94 | 0.217 | 0.022 | **0.001** |
| *L. sativum* | 97 | 91 | 96 | 0.058 | 0.693 | 0.130 |
| **Leaves** | | | | | | |
| Species | %Earth | %moon | %Mars | Earth-moon | Earth-Mars | moon-Mars |
| *A. montana* | 4 | 1 | 5 | 0.151 | 0.721 | 0.076 |
| *S. arvensis* | 2 | 5 | 27 | 0.237 | **0.000** | **0.000** |
| *U. dioica* | 47 | 18 | 59 | **0.001** | 0.153 | **0.000** |
| *C. palustre* | 53 | 36 | 58 | 0.054 | 0.562 | 0.014 |
| *S. reflexum* | 87 | 61 | 86 | **0.000** | 0.847 | **0.000** |
| *F. rubra* | 90 | 70 | 92 | **0.000** | 0.618 | **0.000** |
| *V. sativa sativa* | 31 | 0 | 20 | **0.000** | 0.062 | **0.000** |
| *L. angustifolius* | 26 | 2 | 25 | **0.000** | 0.865 | **0.000** |
| *M. officinalis* | 40 | 37 | 59 | 0.728 | 0.036 | 0.016 |
| *L. pendunculatus* | 73 | 51 | 73 | **0.002** | 1.000 | **0.002** |
| *S. lycopersicum* | 57 | 43 | 84 | 0.139 | **0.003** | **0.000** |
| *S. cereale* | 71 | 61 | 83 | 0.151 | 0.057 | **0.002** |
| *D. carota* | 72 | 50 | 90 | **0.006** | **0.006** | **0.000** |
| *L. sativum* | 89 | 74 | 94 | **0.004** | 0.192 | **0.000** |
| **Flowers** | | | | | | |
| Species | %Earth | %moon | %Mars | Earth-moon | Earth-Mars | moon-Mars |
| *A. montana* | 0 | 0 | 0 | - | - | - |
| *S. arvensis* | 0 | 1 | 21 | 0.238 | **0.000** | **0.000** |
| *U. dioica* | 0 | 0 | 0 | - | - | - |
| *C. palustre* | 0 | 0 | 0 | - | - | - |
| *S. reflexum* | 0 | 0 | 0 | - | - | - |
| *F. rubra* | 0 | 0 | 0 | - | - | - |
| *V. sativa sativa* | 0 | 0 | 0 | - | - | - |
| *L. angustifolius* | 0 | 0 | 0 | - | - | - |
| *M. officinalis* | 0 | 0 | 0 | - | - | - |
| *L. pendunculatus* | 0 | 0 | 0 | - | - | - |
| *S. lycopersicum* | 0 | 0 | 0 | - | - | - |
| *S. cereale* | 25 | 17 | 60 | 0.141 | **0.000** | **0.000** |
| *D. carota* | 0 | 0 | 0 | - | - | - |
| *L. sativum* | 26 | 7 | 50 | **0.003** | **0.004** | **0.000** |
| **Alive after 50 days** | | | | | | |
| Species | %Earth | %moon | %Mars | Earth-moon | Earth-Mars | moon-Mars |
| *A. montana* | 8 | 2 | 8 | 0.040 | 1.000 | 0.040 |
| *S. arvensis* | 3 | 2 | 28 | 0.648 | **0.000** | **0.000** |
| *U. dioica* | 40 | 5 | 56 | **0.000** | 0.050 | **0.000** |
| *C. palustre* | 46 | 18 | 54 | **0.001** | 0.324 | **0.000** |
| *S. reflexum* | 85 | 58 | 86 | **0.000** | 0.842 | **0.000** |
| *F. rubra* | 86 | 48 | 91 | **0.000** | 0.382 | **0.000** |
| *V. sativa sativa* | 32 | 0 | 22 | **0.000** | 0.096 | **0.000** |
| *L. angustifolius* | 33 | 10 | 32 | **0.002** | 0.900 | **0.003** |
| *M. officinalis* | 43 | 22 | 53 | 0.021 | 0.289 | **0.001** |
| *L. pendunculatus* | 71 | 6 | 74 | **0.000** | 0.674 | **0.000** |
| *S. lycopersicum* | 56 | 19 | 79 | **0.000** | **0.005** | **0.000** |
| *S. cereale* | 71 | 53 | 88 | 0.026 | 0.013 | **0.000** |
| *D. carota* | 71 | 20 | 89 | **0.000** | 0.038 | **0.000** |
| *L. sativum* | 85 | 30 | 94 | **0.000** | 0.221 | **0.000** |
