# Supplementary material for: Can Plants Grow on Mars and the Moon: A Growth Experiment on Mars and Moon Soil Simulants
Source: PLoS One. 2014 Aug 27;9(8):e103138. doi: 10.1371/journal.pone.0103138 (PMC4146463; doi:10.1371/journal.pone.0103138)
Supplement: File S1 — Photos of the experiment. (DOCX) [file pone.0103138.s003.docx]

Photos of the experiment.


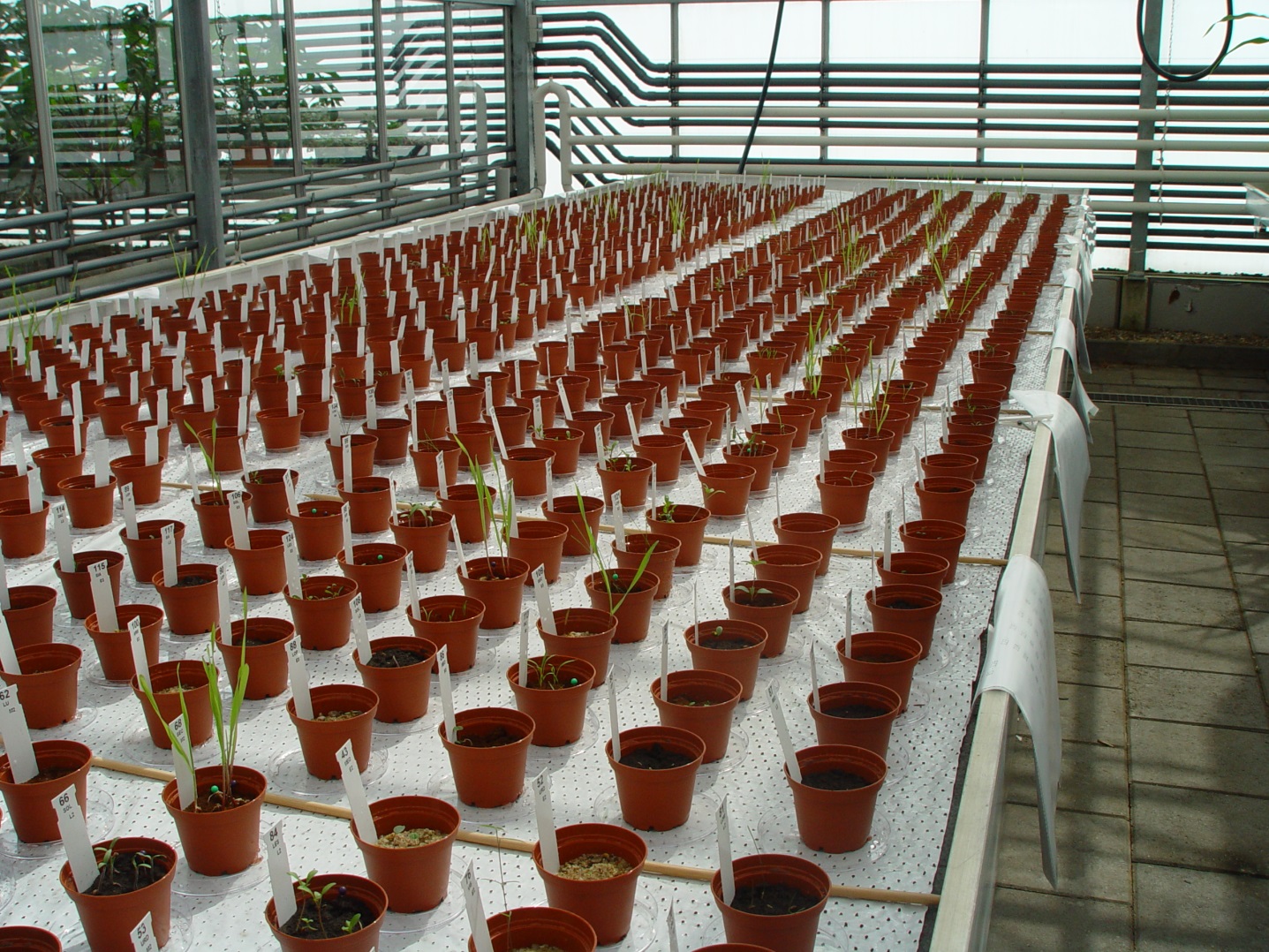
Photo 1. Overview of the experimental set up.


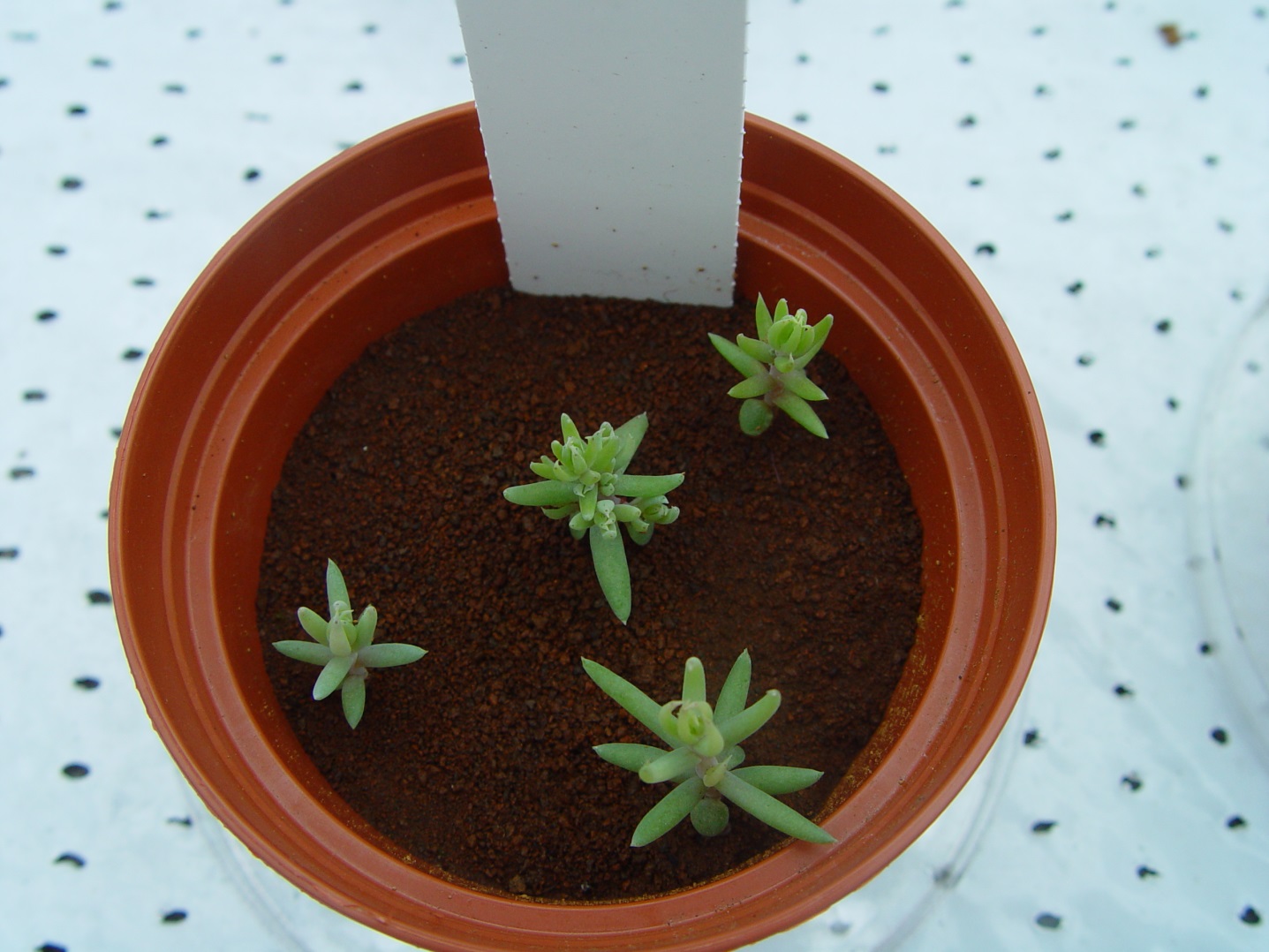


Photo 2. Reflexed stonecrop on artificial Martian soil.


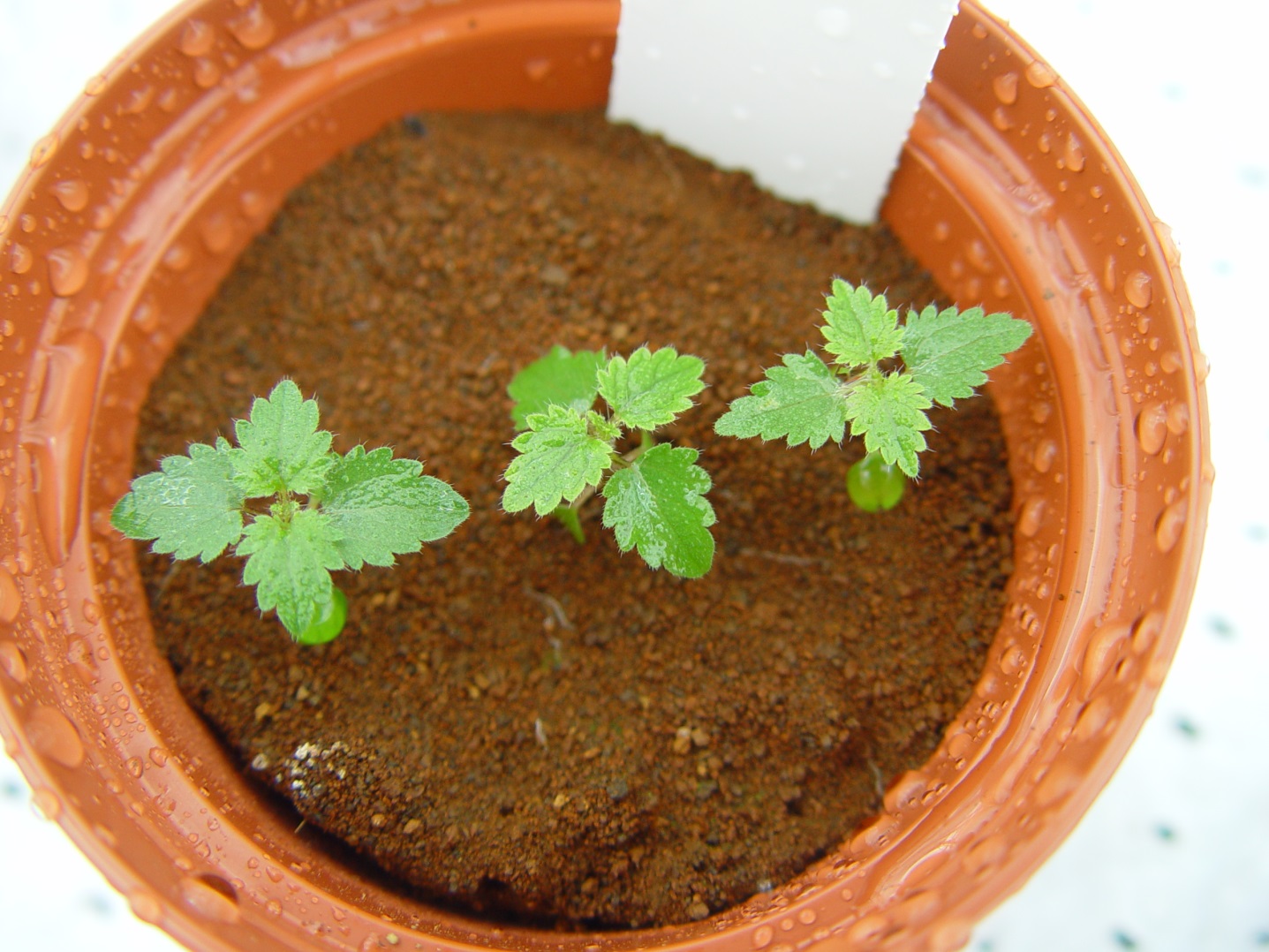


Photo 3. Stinging nettle on artificial Martian soil.


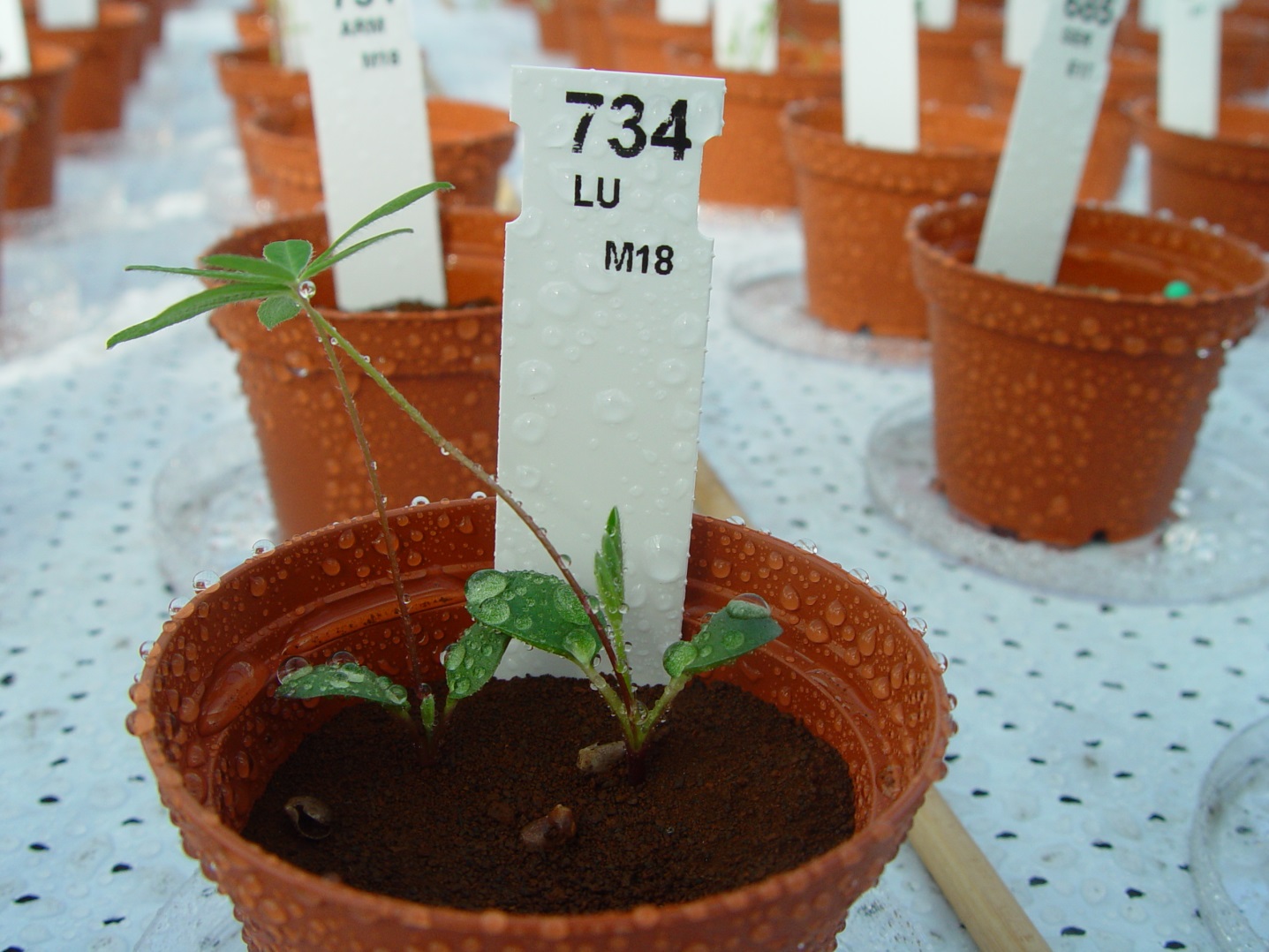


Photo 5. Lupin on artificial Martian soil.


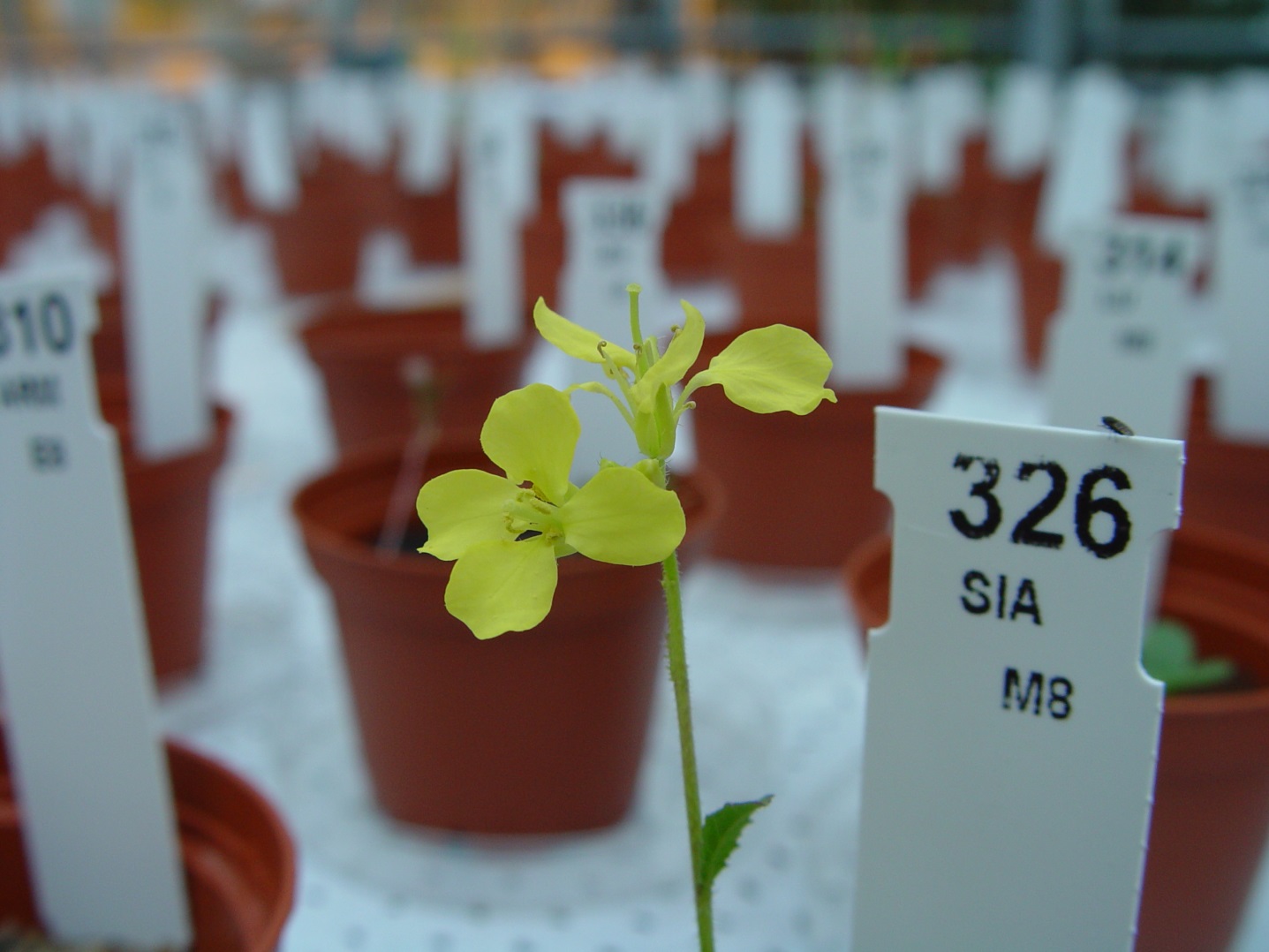


Photo 6. Field mustard flowering on artificial Martian soil.


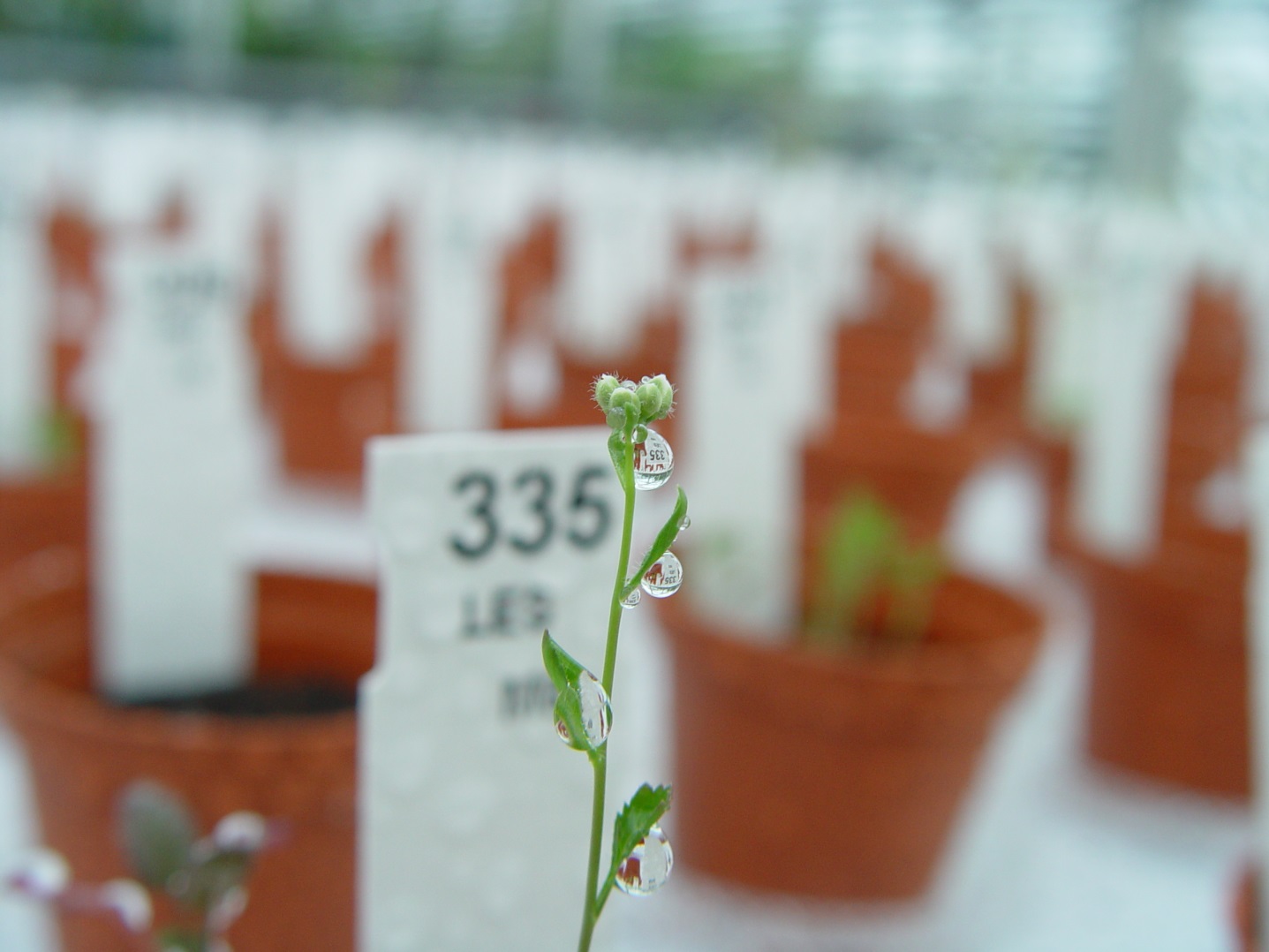
Photo 7. Cress on artificial Martian soil.


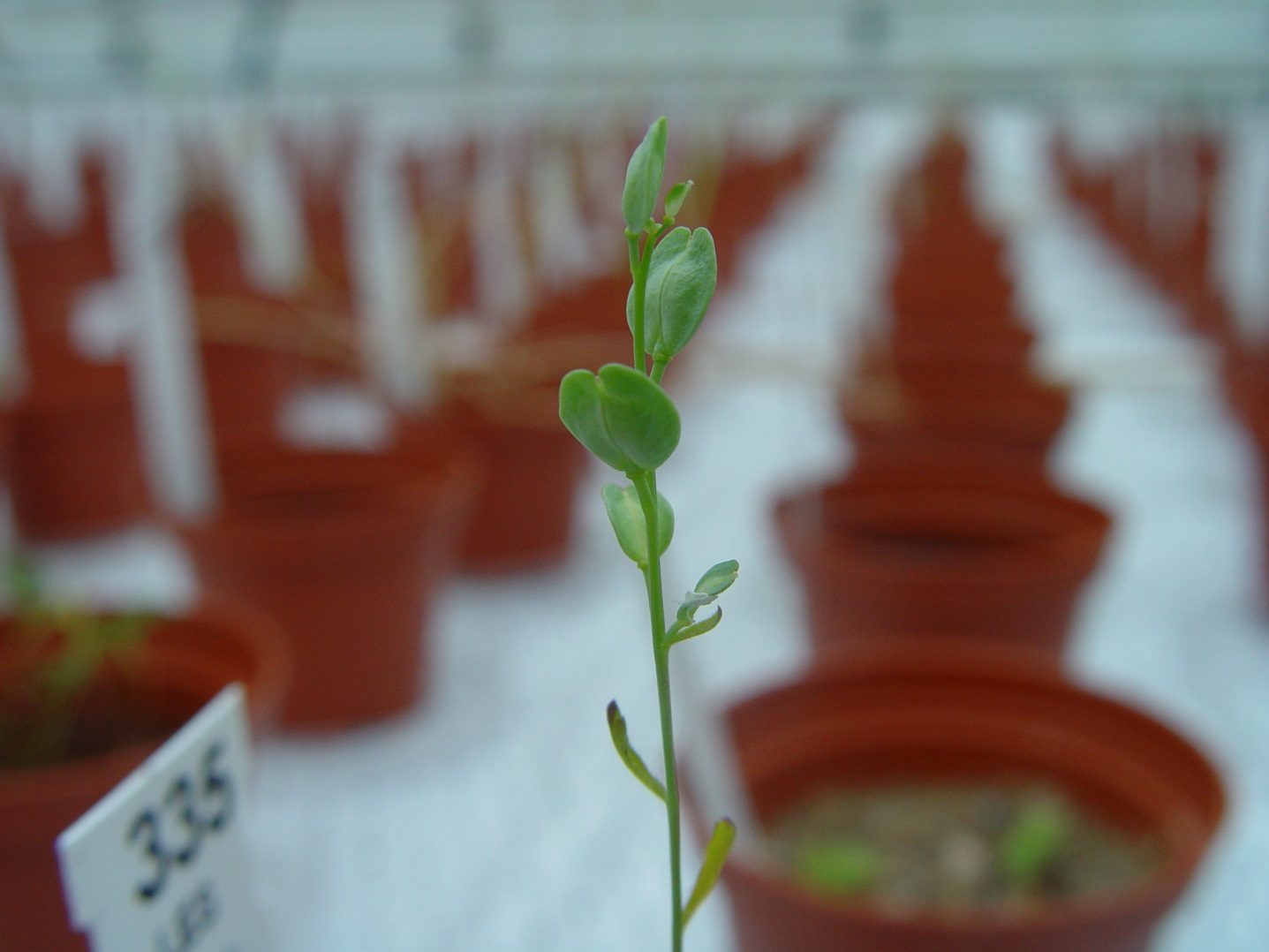


Photo 8. Cress with seeds on artificial Martian soil.


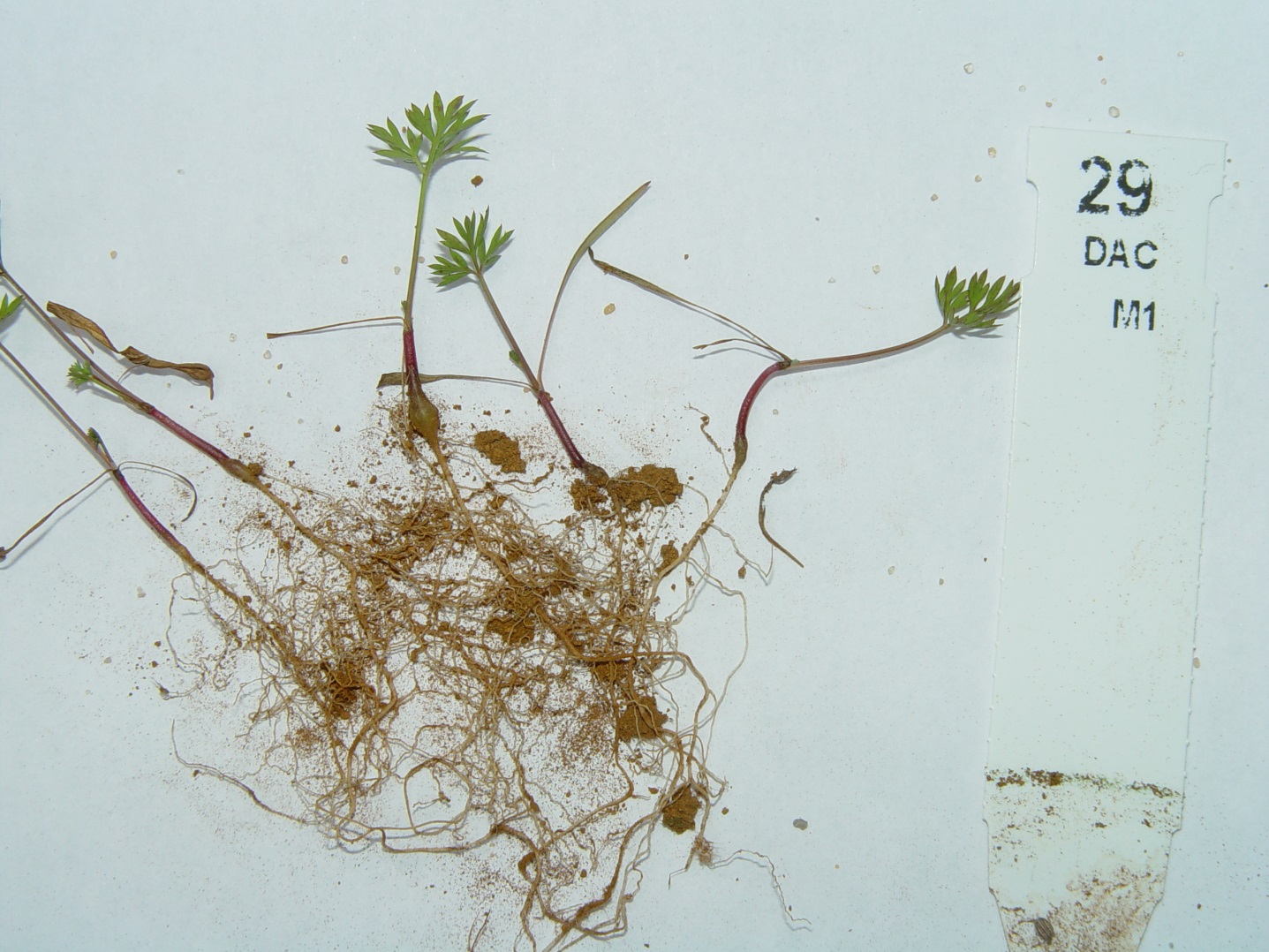


Photo 9. Carrots after harvest on artificial Martian soil, Note the small start of the thick parts of the root, forming the eatable part.


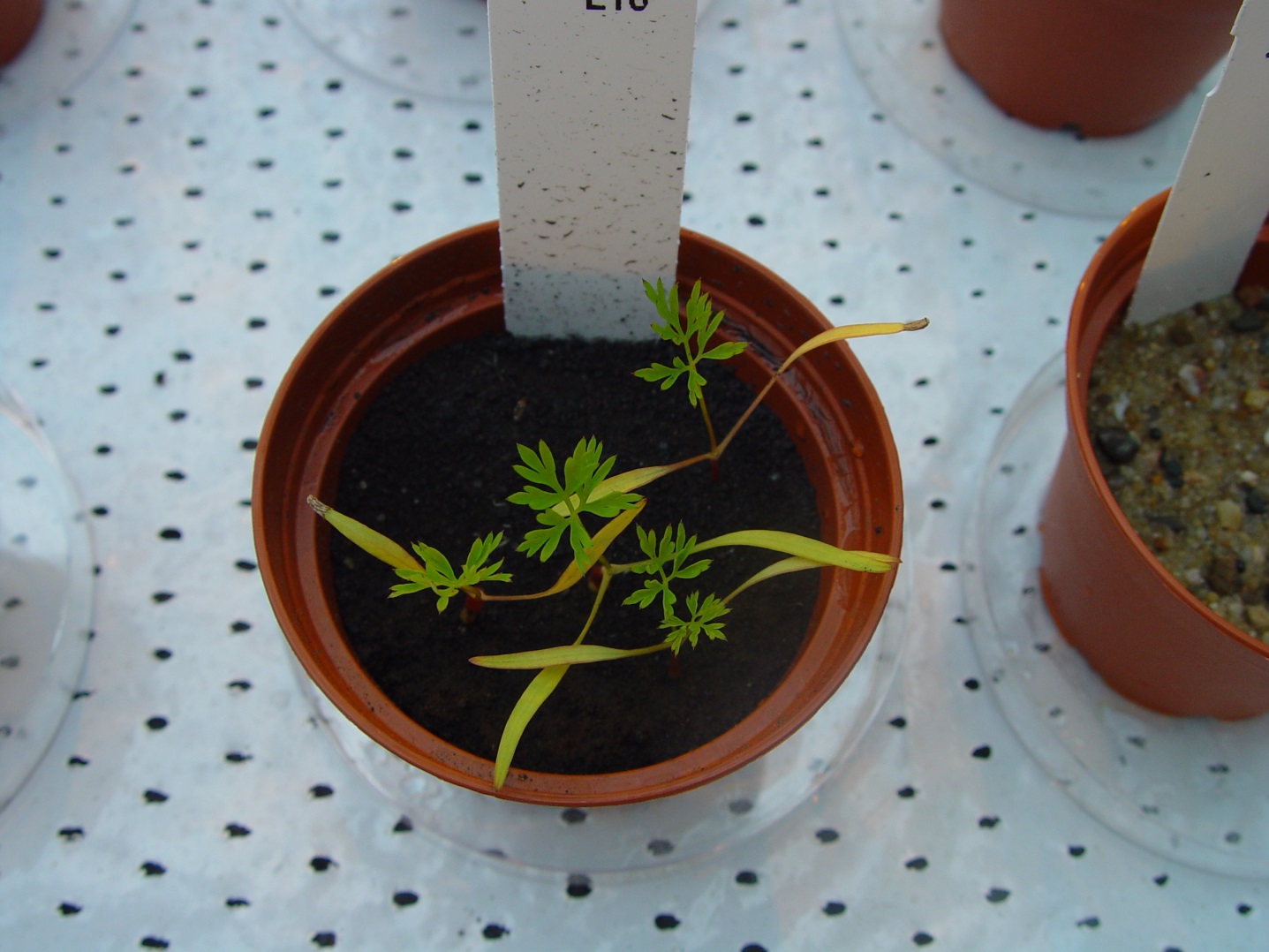


Photo 10. Carrot on artificial Moon soil.
